# Identification of Alkaloids from *Corydalis* *yanhusuo* W. T. Wang as Dopamine D_1_ Receptor Antagonists by Using CRE-Luciferase Reporter Gene Assay

**DOI:** 10.3390/molecules23102585

**Published:** 2018-10-10

**Authors:** Lehao Wu, Weiyue Zhang, Xin Qiu, Chaoran Wang, Yanfang Liu, Zhiwei Wang, Yang Yu, Richard D. Ye, Yan Zhang

**Affiliations:** 1 School of Pharmacy, Shanghai Jiao Tong University, Shanghai 200240, China; wulehaogo@sjtu.edu.cn (L.W.); zhangweiyue@sjtu.edu.cn (W.Z.); qiux94@sjtu.edu.cn (X.Q.); yuyang2011@sjtu.edu.cn (Y.Y.); RichardYe@umac.mo (R.D.Y.); 2 Key Lab of Separation Science for Analytical Chemistry, Dalian Institute of Chemical Physics, Chinese Academy of Sciences, Dalian 116023, China; wcrpj_505@dicp.ac.cn (C.W.); lyf-121katy@163.com (Y.L.); 3 DICP-CMC Innovation Institute of Medicine, Taizhou 225300, China; wangzhiwei@castim.cn; 4 State Key Laboratory of Quality Research in Chinese Medicine, Institute of Chinese Medical Sciences, University of Macau, Macau 999078, SAR, China

**Keywords:** *Corydalis yanhusuo* W. T. Wang, luciferase reporter gene assay, D_1_ receptor, FLIPR

## Abstract

*Corydalis yanhusuo* W. T. Wang (*C. yanhusuo*) has been traditionally used for drug addiction and pain relief in China. In our previous study, we showed that the extract of *C. yanhusuo* blocks dopamine receptors, demonstrating that its pharmacological activities are mostly due to the antagonistic effects of some of its components at dopamine receptors. As part of our ongoing project on *C. yanhusuo*, the aim of the present study is to establish a high-throughput and low-cost screening assay system and test the abilities of the isolated alkaloids from *C. yanhusuo* to inhibit dopamine-induced dopamine D_1_ receptor activity. By using our established cyclic adenosine monophosphate (cAMP)-response element (CRE)-luciferase reporter gene assay system, we identified eight alkaloids from *C. yanhusuo* with D_1_ receptor antagonistic activities. We next validated the activities of these compounds using fluorometric imaging plate reader (FLIPR) assay by measuring the intracellular Ca^2+^ change. Six out of eight compounds, including tetrahydropalmatine, corydaline, 13-methyldehydrocorydalmine, dehydrocorybubine, dehydrocorydaline, and columbamine, can be confirmed for their inhibitory activities. The dopamine-receptor-antagonistic effects of four compounds, including 13-methyldehydrocorydalmine, dehydrocorydaline, columbamine, and corydaline, are reported for the first time. The present study provides an important pharmacological basis to support the traditional use of *C. yanhusuo* in China.

## 1. Introduction

Dopamine receptors belong to the superfamily of G protein–coupled receptors (GPCR). To date, five different subtypes of dopamine receptors (D_1–5_) have been cloned from different species [1]. D_1_ and D_5_ subtypes generally are coupled to G_s_ proteins, which increase intracellular cyclic adenosine monophosphate (cAMP) levels and is categorized into D_1_-like family; D_2_, D_3_, and D_4_ are coupled to G_i/o_, which decrease intracellular cAMP levels and are categorized into D_2_-like family [2]. Dopamine receptors are involved in diverse neurological processes, including motivation, pleasure, memory, and learning [3]. Abnormal dopamine receptor signalling is implicated in several neuropsychiatric disorders, and dopamine receptors are common therapeutic targets for neuropsychiatric drugs [4]. Most antipsychotics have dopamine receptor antagonistic properties, and some of the D_1_-like receptor antagonists are used for the treatment of drug addiction [5].

Current methods employed in the assessment of dopamine receptor activation or antagonism are mainly through determining the production of second messengers, such as cAMP or Ca^2+^, depending on the type of G protein to which the receptor is coupled [6,7,8]. The cost, efforts, and specialized instrumentation required for these second messenger assays limit their feasibility or large-scale screening, especially in academic laboratories [9]. Over the years, the reporter gene assay remains a popular assay format due to its wide dynamic range, robustness, cost-effectiveness, and ease to set up [10]. A reporter gene assay system typically consists of specific responsive elements placed upstream of a minimal promoter, which together control the expression of an easily measurable reporter protein, such as luciferase. The coupling of these reporter genes with enhancer elements, such as cAMP-response elements (CREs), serum-response elements (SREs), or nuclear factor of activated T cells (NFAT), leads to second-messenger-depending gene expression following receptor activation [10,11].

*Corydalis yanhusuo* W. T. Wang (*C. yanhusuo*) has been traditionally used for drug addiction and pain relief in China for centuries [12]. It has been demonstrated that its biological properties are mainly associated with *C. yanhusuo* alkaloids. In our previous study, we found dehydrocorybulbine, an alkaloid isolated in *C. yanhusuo*, as well as its crude extract exhibit antagonistic activities at dopamine receptors [13,14]. We hypothesize that *C. yanhusuo* may contain previously unidentified alkaloids acting on dopamine receptors that may also contribute to its pharmacological effects.

As part of our ongoing project of systematically investigating *C. yanhusuo*, we initiated a comprehensive study in an attempt to identify new dopamine receptor antagonists from *C. yanhusuo*. Here, we aim to develop a low-cost luciferase reporter gene assay to identify dopamine D_1_ receptor antagonists from *C. yanhusuo* and foster a better understanding of the traditional use of *C. yanhusuo*.

## 2. Results

### 2.1. Development of Reporter Gene Assay for D_1_ Receptor Activation

The cDNA plasmids of dopamine D_1_ receptor and CRE/Luci were stably co-transfected into human embryonic kidney-293 cells (HEK293) cells (HEK-D_1_/CRE/Luc2P). Activation of D_1_ receptors leads to the elevation of cAMP and activates the cAMP response element (CRE) and therefore induces the expression of the reporter gene luciferase. We used the natural ligand dopamine as a positive control to select the cell colony and to optimize and characterize the HEK-D_1_/CRE/Luc2P cell system.

**Figure 1:**
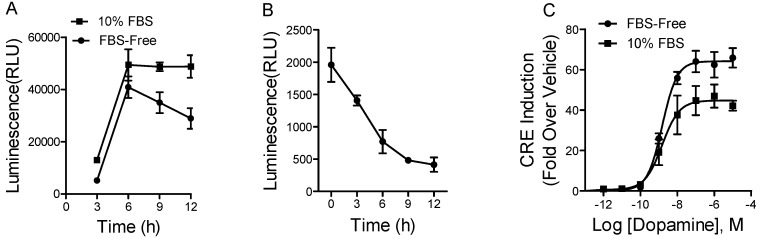
The optimization of the luciferase assay. (**A**) The effects of fetal bovine serum (FBS) on 1 μM dopamine-induced firefly luciferase activity in HEK-D_1_/CRE/Luc2P cells; (**B**) The time course of the background induced by vehicle in HEK-D_1_/CRE/Luc2P cells; (**C**) The dose-dependent response of dopamine in HEK-D_1_/CRE/Luc2P cells with or without FBS in the medium. The incubation time for dopamine is 6 h. Data of (C) were analyzed with Prism 5 using nonlinear regression. The results are expressed as the mean ± SD (*n* = 3).

We carried out a series of experiments to optimize the assay. First, we examined the effects of fetal bovine serum (FBS) on D_1_ receptor activation at different time points by using 1 μM of dopamine. As shown in Figure 1A, dopamine showed differential but robust responses at different time points. Dopamine gave a slightly higher response at all time points in the presence of FBS in the medium compared to that in the FBS-starved medium. In order to avoid the disruption of serum in some cases, we chose to use the FBS-starved medium for the further tests. As seen in Figure 1A, the luciferase activity reached the maximal response in 6 h when the cells were induced by 1 μM of dopamine. The background (induced by vehicle) was decreased with longer incubation time (Figure 1B). The 6-h incubation with the vehicle control gave a slightly higher background signal compared to 9- and 12-h incubation. Therefore, we used a 6-h incubation time as the compromised condition for all our experiments, unless otherwise indicated.

Next, we tested the dose-response curve of the natural ligand dopamine to the D_1_ receptor under the optimized conditions, including incubation in FBS-starved medium for 6 h. As shown in Figure 1C, dopamine showed robust agonist effects. The half-maximal effective concentration (EC_50_) value derived from nonlinear curve-fitting analysis is 1.5 nM, which is comparable to the published data [15,16]. The dynamic range (fold of induction) is from 1 to 40.

### 2.2. Amenable for High-Throughput Screening (HTS)

**Figure 2:**
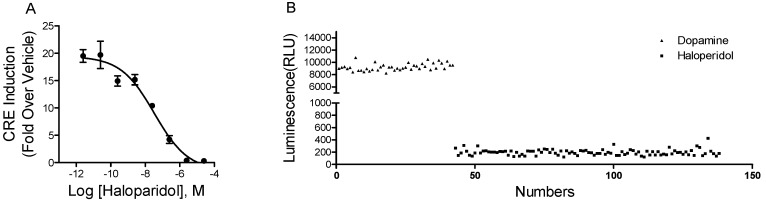
The characterization of the luciferase reporter gene assay system. (**A**) The dose-dependent inhibitory effects of haloperidol against dopamine D_1_ receptor. Data were analyzed with Prism 5 using nonlinear regression. The results are expressed as the mean ± SD (*n* = 3); (**B**) The calculation of the Z′-factor in a 96-well plate format of this assay. Cells were treated with haloperidol (2 μM) or vehicle for 1 h, then incubated with dopamine (2 nM) for another 6 h.

To determine whether the luciferase reporter gene assay is suitable for HTS of D_1_ receptor antagonists, we examined the inhibitory effects of haloperidol, a known nonselective dopamine receptor antagonist, at various concentrations against the stimulation of 2 nM of dopamine (Figure 2A). The half-maximal inhibitory concentration (IC_50_) of haloperidol in the luciferase reporter gene assay was 34 nM, which is consistent with the published data [17].

Z’-factor is one of the major statistical parameters commonly used to evaluate this assay performance in HTS [18]. We calculated the Z′-factor in a 96-well plate format (Figure 2B). According to the formula, the closer the Z′-factor is to 1, the better the assay quality is. In general, Z′-factor values greater than 0.5 are acceptable for HTS. In this assay, the Z′-factor value was 0.79, markedly higher than 0.5. Thus, the developed luciferase reporter gene assay is robust and can be used for HTS of D_1_ receptor antagonists.

### 2.3. Screening of D_1_ Dopamine Receptor Antagonists with Luciferase Reporter Gene Assay

**Figure 3:**
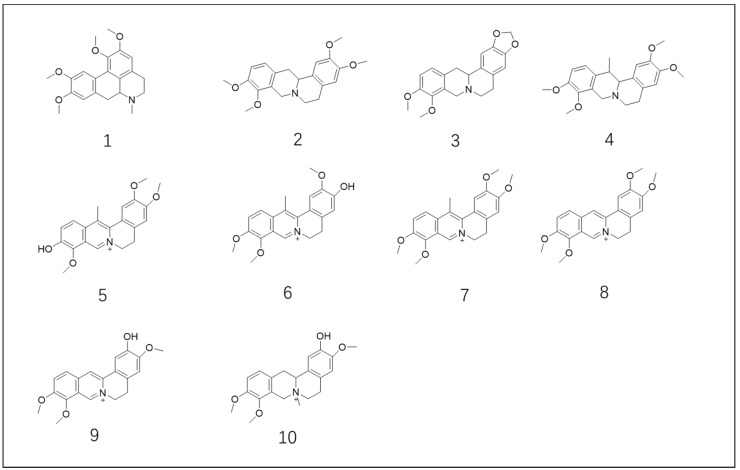
Structures of the isolated compounds from *Corydalis yanhusuo* W. T. Wang (*C. yanhusuo*).

**Figure 4:**
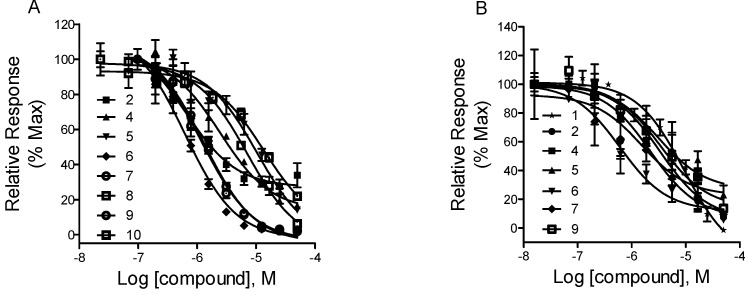
Pharmacological profiles of the compounds isolated from *C. yanhusuo* on dopamine D_1_ receptor using luciferase reporter gene (**A**) or FLIPR assay (**B**). (**A**) The dose-dependent inhibition of the compounds in the presence of dopamine (2 nM) in HEK-D_1_/CRE/Luc2P cells. The firefly luciferase activity was measured. (**B**) The dose-dependent inhibition of the compounds in the presence of dopamine (500 nM) in HEK-D_1_/G_α15_ cells. The Ca^2+^ mobilization was measured by FLIPR. Relative responses were calculated by setting each value without tested compounds (dopamine alone) at 100%. All data were analyzed with Prism 5 using nonlinear regression. The results are expressed as the mean ± SD (*n* = 3).

Ten alkaloids (as listed in Table 1 and Figure 3) isolated from *C. yanhusuo* were tested at an initial single screening concentration of 12.5 μM. By setting an activity threshold of 50%, eight compounds, including tetrahydropalmatine (**2**), corydaline (**4**), 13-methyl-dehydrocorydalmine (**5**), dehydrocorybulbine (**6**), dehydrocorydaline (**7**), palmatine (**8**), columbamine (**9**), and *N*-methyltetrahydrocolumbamine (**10**), were selected for further study. Concentration–response experiments were then performed on these eight primary hit compounds to determine the IC_50_ values. As shown in Figure 4A, all eight compounds showed typical concentration-dependent antagonist responses. Among the eight compounds, compounds **2** and **10** showed partial antagonist effects because they were not able to inhibit the dopamine response completely even at high concentrations. The IC_50_ values of the eight compounds are listed in Table 1. The best inhibitory compounds, as measured by the lowest IC_50_ values, are dehydrocorybulbine (compound **6**) and tetrahydropalmatine (compound **2**).

HEK293 cells stably expressing cDNA plasmid of CRE/Luciferase (HEK/CRE/Luc2P) were established as the negative control cells to test the specificity to the D_1_ receptor for the compounds. Forskolin, which can activate the enzyme adenylyl cyclase, was used in this study to increase intracellular levels of cAMP with an EC_50_ value of 1.2 μM, as shown in Figure S1. We then examined whether the primary hit compounds could nonspecifically inhibit the forskolin-stimulated (1 μM) response. As shown in Figure S2, high doses of compounds **5** and **8** decreased the cAMP levels in HEK/CRE/Luc2P cells by 25%, while others did not produce significant changes, indicating that these two compounds may also act at some signal molecules in the cAMP/CRE/Luciferase pathways in HEK cells.

### 2.4. Fluorescent Ca^2+^ Mobilization Assay

We used a fluorescent Ca^2+^ mobilization assay to further evaluate the hit compounds obtained from the luciferase reporter gene assay screens. The cDNA plasmids of D_1_-like receptors were stably transfected into HEK 293T cells. With the coexpression of G_α15_ protein, D_1_ receptors can be redirected to mediate intracellular calcium mobilization upon stimulation. Calcium levels were monitored using fluorometric imaging plate reader (FLIPR). The assay can also distinguish potential antagonists from agonists. Dopamine stimulated the Ca^2+^ mobilization in HEK 293T cell expressing human D_1_ receptors and G_α15_ with an EC_50_ value of 580 nM. As shown in Figure 4B, six compounds, including compounds **2**, **4**, **5**, **6**, **7**, and **9**, displayed concentration-dependent antagonist responses. The IC_50_ values were similar to those measured by luciferase assay. Compounds **8** and **10** showed weak inhibitory effects because they inhibited the dopamine D_1_ receptor only at very high concentrations. 

## 3. Discussion and Conclusions

GPCRs mediate various biological functions in response to a variety of ligands. GPCRs are involved in many signal transduction pathways and have been associated with numerous human diseases such as schizophrenia [19], cancers [20], and atherosclerosis [21]. Thus, these receptors have been used as therapeutic targets for a large number of drugs. The dopamine D_1_ receptor, which belongs to GPCRs, plays an important role in pain [14], hypertension [22], and the regulation of sodium and body volume homeostasis [23]. Several alkaloids isolated from *C. yanhusuo* have been found to act on dopamine receptors, including tetrahydropalmatine, dehydrocorybulbine, and other isoquinoline alkaloids [14,24,25]. In our ongoing project on the systematic investigation of *C. yanhusuo*, we have purified a series of alkaloids from *C. yanhusuo* extract. In order to determine their potential effects on dopamine receptors, we set to examine the antagonistic activity on the dopamine D_1_ receptor by using our established luciferase reporter gene assay system.

The reporter gene system is a technology where a reporter gene is synthesized in response to the specific signalling cascade under investigation. The activation or inactivation of the signalling cascade can be measured by monitoring the reporter protein expression by its enzymatic activities linked with a variety of colorimetric, fluorescent, or luminescent readouts. For GPCR assays, the activity of a receptor coupled to a G_αs_ protein is monitored by using a CRE positioned upstream of a reporter gene. In this study, we expressed the D_1_ receptor and CRE in pGL4.29 vectors containing luciferase reporter genes. The activation of D_1_ receptors leads to the activation of adenylyl cyclase and the increase of the intracellular concentration of cAMP. The cAMP second messenger system ultimately activates CRE and induces luciferase gene expression. Luciferase catalyzes a two-step process involving the conversion of D-luciferin substrate to oxyluciferin and yields emissions of yellow/green light (550–570 nm) [26]. We established this bioluminescent reporter assay to identify the antagonists of D_1_ receptors by measuring enzymatic activity in response to changes in the levels of cAMP. Compared to other GPCR assays, the luciferase reporter gene assay system is cost-effective, easy to handle, sensitive, and suitable for a large-scale culture and automation. 

Using this CRE-luciferase reporter gene assay system, we identified eight alkaloids from *C. yanhusuo* in the D_1_ receptor antagonist tests. We next validated these compounds with a FLIPR assay by measuring the intracellular Ca^2+^ change. Six out of eight compounds can be confirmed for their inhibitory activities. Two out of eight compounds, compounds **8** and **10**, displayed very poor antagonistic effects on dopamine D_1_ receptors in the FLIPR assay. Glaucine (compound **1**) demonstrated a significant blockade on D_1_ receptors in the FLIPR assay, while in luciferase reporter gene assay, it exhibited a biphasic curve. As shown in Figure S3, glaucine showed antagonistic activity in a low-dose range and agonistic activity at high doses. This may be explained by the fact that high doses of glaucine may interfere with other cell mechanisms necessary for the production of luciferase reporter, since the luciferase activity did increase when glaucine alone was tested in HEK-D_1_/CRE/Luc2P (Figure S3). Compounds **6** and **7** displayed fairly steeper dose–response curves in the luciferase reporter gene assay than those in the FLIPR assay (Figure 4). The exact reason for the steeper curves is unclear, but it might be due to the nonselective effects of these two compounds on dopamine D_1_ receptors in the luciferase reporter gene assay. Because calcium mobilization occurs early in the signalling pathway, the FLIPR assay measures the early events of GPCR activation, including agonist–GPCR binding, G-protein activation, and second messenger release [27]. However, the reporter gene assay reports the late response of GPCR activation because a more complete signalling pathway is needed for the activation of the target gene. In our established luciferase reporter gene system, when the level of intracellular second messenger cAMP increased, cAMP-dependent protein kinase A (PKA) was activated, and subsequently, the CRE-binding protein (CREB) was phosphorylated. This enabled CREB to bind to CRE in the promoter of the luciferase reporter gene, thus increasing its transcription [11]. Since the reporter gene assay measures the activation of entire effectors in the signalling cascades, there is much scope for the tested compounds to interfere with the signalling pathway. If a compound under study interacts with downstream components of the signalling cascade, the reporter readout reflects a mixed activity at the receptor and at a point elsewhere in the signalling cascade. This is also the reason why the luciferase reporter gene assays occasionally give false-positive results. Therefore, it is necessary to validate the hit compounds primarily identified in a reporter gene assay in a second assay format or against more GPCR assay systems constructed simultaneously in practically the same way. Using other GPCR assay systems as controls, we were able to select compounds that interact only with the target GPCR. The false-positive rate can be partially resolved by the coexpression of a constitutively expressed internal control (*Renilla luciferase*) [28]. On the other hand, when the antagonism of compounds is tested, using a selective GPCR agonist would have been helpful to find the specific GPCR inhibitors and simplify data interpretations.

Tetrahydropalmatine (compound **2**) and dehydrocorybulbine (compound **6**) have been reported to be the primary analgesic components in *C. yanhusuo* and act on dopamine receptors [14,29]. In the present study, both of these two compounds showed the highest antagonistic potency on dopamine D_1_ receptors.

Dehydrocorydaline (compound **7**), an alkaloidal component, has been recently reported to exert analgesic [30], anti-inflammatory [31], and antitumor effects [32]. Also, it has been found to act on opioid receptors [30]. However, very limited information is available regarding its action on dopamine receptors. Therefore, we are the first to report its antagonistic activity on dopamine D_1_ receptors. 

13-methyl-dehydrocorydalmine (compound **5**) was originally isolated from the tuber of *C. yanhusuo* in 2008 [33]. However, this was not followed by further investigation on this compound. In the present study, we reported for the first time that 13-methyl-dehydrocorydalmine exhibits inhibitory activity against dopamine D_1_ receptors.

Columbamine (compound **9**) and corydaline (compound **4**) are isoquinoline alkaloids which have been found to bind to dopamine D_1_ receptors [25]. The present study reveals that these two compounds are antagonists of dopamine D_1_ receptors.

Given the limited number of available compounds, it is difficult to derive a structure–activity relationship. However, the -CH_3_ group at C_13_ and the position of the -OH group appear to be important for the activity of quaternary ammonium alkaloids because compounds **5**, **6**, **7**, **8**, and **9** exhibited differences in antagonist potencies, although they have a highly similar structure.

In conclusion, by using our established CRE-luciferase reporter gene and FLIPR assay systems, we identified six alkaloids from *C. yanhusuo* with D_1_ receptor antagonistic activities. The dopamine-receptor-antagonistic effects of four compounds, including 13-methyldehydrocorydalmine, dehydrocorydaline, columbamine, and corydaline, are reported for the first time. The present study demonstrated that alkaloids are at least partially active constituents of *C. yanhusuo* extracts that antagonize dopamine D_1_ receptors. Further studies are required to clarify their activity against other dopamine receptor subtypes. This study provides an important pharmacological basis to support the traditional use of *C. yanhusuo* in China.

## 4. Materials and Methods

### 4.1. Materials

Human embryonic kidney-293 cells (HEK293) were cultured in Dulbecco’s Minimum Essential Medium (DMEM) supplemented with 10% fetal bovine serum (FBS). Dopamine D_1_ receptor gene cloned in pcDNA 3.1 was kindly provided by Dr. Olivier Civelli (University of California, Irvine). pGL4.29 (luc2P/CRE/Hygro) luciferase reporter gene was purchased from Promega (Madison, WI, USA). Lipofectamine 2000 was purchased from Invitrogen (Caelsbad, CA, SUA). Dopamine was purchased from Sigma (St. Louis, MO, USA). Fluo-4 AM was purchased from Molecular Probes Inc. (Eugene, OR, USA).

### 4.2. Plant

*C. yanhusuo* was collected from Dongyang County (Zhejiang, China). The herb was authenticated by the Institute of Medication, Xiyuan Hospital of China Academy of Traditional Chinese Medicine. The extraction procedure and compound isolation were performed as previously reported [34,35,36].

### 4.3. Creation of the Stable Cell Line

The pGL4.29 (CRE/luc2P//Hygro) luciferase reporter gene was stably co-transfected with dopamine D_1_ receptor plasmid or was stably transfected individually in HEK293 cells. Transfection was carried out with lipofectamine 2000 reagent using the protocol provided by Promega. Stable cells expressing CRE-Luciferase and D_1_ receptor were selected in the presence of 700 μg/mL G418 and 200 μg/mL hygromycin for 10–14 days. Stable cells expressing CRE-Luciferase were singly selected in the presence of 200 μg/mL hygromycin. The obtained stable cells were then cultured with 350 μg/mL G418 and 100 μg/mL hygromycin or only 100 μg/mL hygromycin to maintain the selective growth.

### 4.4. Luciferase Reporter Gene Assay

The examination of CRE-luciferase activity was performed by a modification of methods as described previously [37,38]. HEK293 stable cells were seeded in 96-well plates at a density of 1.5 × 10^4^ cells per well. After cells became adherent, they were stimulated in serum-starved DMEM for different hours to explore the most suitable starving time. Cells were then pretreated with compounds for 1 h prior to dopamine stimulation. Afterwards, the cells were lysed with luciferase lysis buffer and the luminescence of the firefly luciferase was measured with the FlexStation 3 (Molecular Devices, Sunnyvale, CA, USA).

### 4.5. Fluorescent Ca^2+^ Mobilization Assay

Fluorescent Ca^2+^ mobilization assay was performed using Flu-4 AM reagent as reported earlier [39]. The compounds were dissolved in dimethyl sulfoxide as stock solution and diluted with fluorometric imaging plate reader (FLIPR) buffer and then automatically added into the cells within 4 s. For agonist tests, the intracellular Ca^2+^ concentration was monitored at 520 nm with the excitation wavelength at 488 nm over a period of 4 min. For antagonist tests, the compounds were first incubated with the cells for 10 min before the addition of dopamine.

### 4.6. Data Processing

All statistical calculations were performed using GraphPad Prism 5.0 (GraphPad, Avenida, CA, USA). Data from each dose-response curve were normalized to the maximal stimulation by dopamine. At least three independent experiments were performed.

## Figures and Tables

**Table 1:** IC_50_ values and maximal inhibition of the compounds for dopamine D_1_ receptor obtained by luciferase assay and fluorometric imaging plate reader (FLIPR).

No.	Compounds (CAS No.)	IC_50_ * (μM) with 95% Confidence Intervals		% Maximal Inhibition Mean ± SD (*N* = 3)	
		Luciferase	FLIPR	Luciferase	FLIPR
**1**	Glaucine	/	9.408	/	101.6 ± 0.8452
	(475-81-0)		(6.568–13.48)		
**2**	Tetrahydropalmatine	0.6437	2.240	72.53 ± 3.9953	92.64 ± 1.7945
	(2934-97-6)	(0.4442–0.9327)	(1.060–4.736)		
**3**	Canadine	/	/	/	/
	(522-97-4)				
**4**	Corydaline	2.457	3.360	83.49 ± 1.4462	90.32 ± 1.9817
	(518-69-4)	(1.681–3.591)	(1.626–6.945)		
**5**	13-methyldehydrocorydalmine	10.32	3.079	85.97 ± 1.7628	77.04 ± 6.6410
	(1126743-67-6)	(6.862–15.53)	(1.109–8.550)		
**6**	Dehydrocorybulbine	0.6209	0.6123	97.52 ± 0.6264	94.38 ± 0.1939
	(59870-72-3)	(0.4753–0.8109)	(0.4268–0.8784)		
**7**	Dehydrocorydaline	1.292	1.527	98.23 ± 0.3301	90.61 ± 0.6541
	(30045-16-0)	(1.005–1.661)	(0.5213–4.473)		
**8**	Palmatine	7.082	/	93.86 ± 0.4367	/
	(3486-67-7)	(5.297–9.469)			
**9**	Columbamine	1.210	3.404	97.50 ± 0.2181	86.43 ± 0.7188
	(3621-36-1)	(0.8992–1.629)	(1.951–5.939)		
**10**	*N*-methyltetrahydrocolumbamine	13.36	/	78.15 ± 1.783	/
	(47528-98-3)	(8.246–21.66)			

* The calculation of IC_50_ values were based on the degree of maximal inhibition.

## Supplementary Materials

Including Figures S1–S3.
