# Supplementary figures and images for: Identification of Alkaloids from Corydalis yanhusuo W. T. Wang as Dopamine D1 Receptor Antagonists by Using CRE-Luciferase Reporter Gene Assay
Source: Molecules. 2018 Oct 10;23(10):2585. doi: 10.3390/molecules23102585 (PMC6222624; doi:10.3390/molecules23102585)

Fig.S1

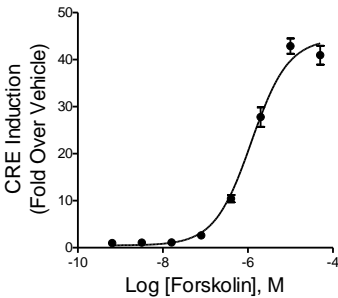

Fig.S2

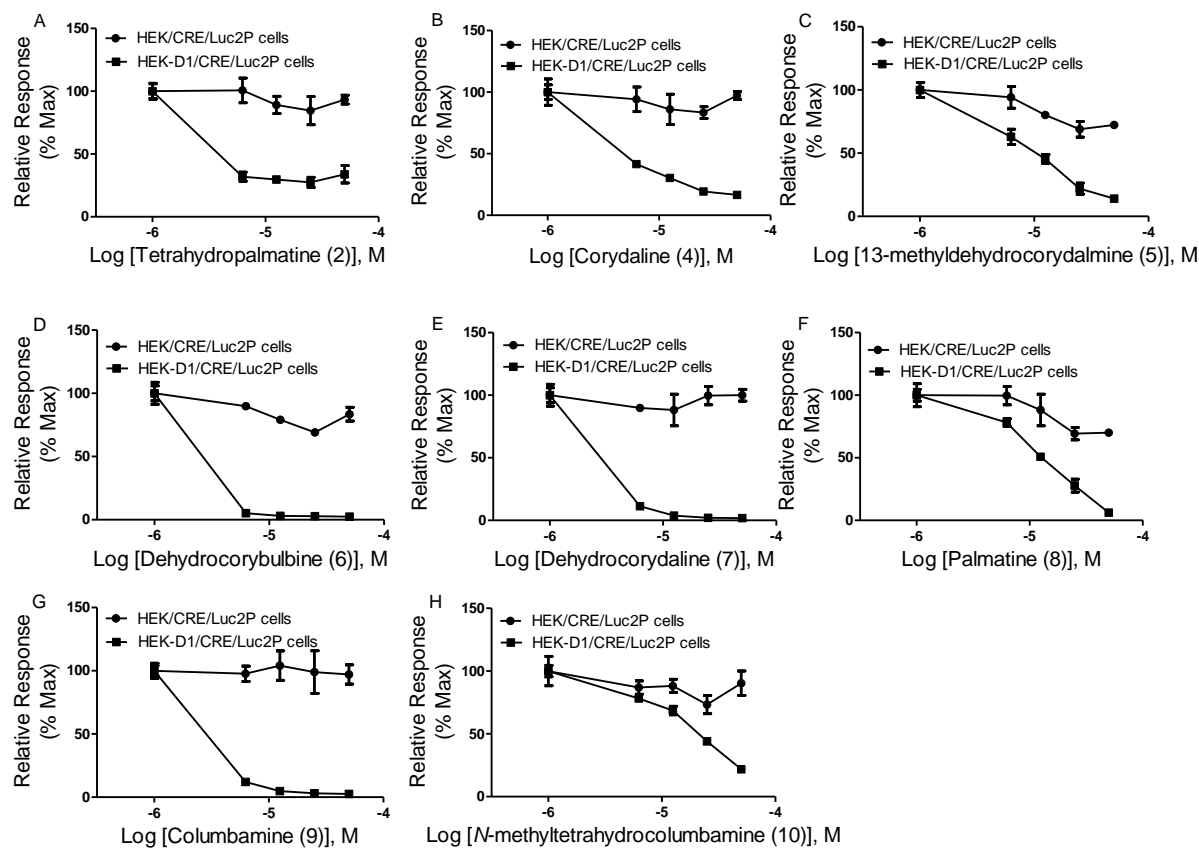

Fig.S3

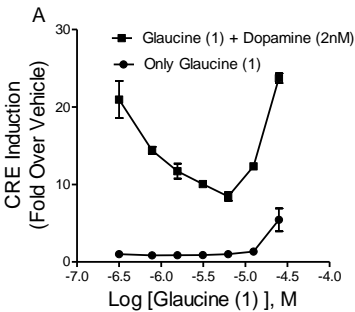

Supplement: Supplementary file 1 [file molecules-23-02585-s001.pdf]
